# Phosphatidylinositol 3-Kinase/Akt and MEK/ERK Signaling Pathways Facilitate Sapovirus Trafficking and Late Endosomal Acidification for Viral Uncoating in LLC-PK Cells

**DOI:** 10.1128/JVI.01674-18

**Published:** 2018-11-27

**Authors:** Mahmoud Soliman, Deok-Song Kim, Jun-Gyu Park, Ji-Yun Kim, Mia Madel Alfajaro, Yeong-Bin Baek, Eun-Hyo Cho, Chul-Ho Park, Mun-Il Kang, Sang-Ik Park, Kyoung-Oh Cho

**Affiliations:** a Laboratory of Veterinary Pathology, College of Veterinary Medicine, Chonnam National University, Gwangju, Republic of Korea; b Chonnam National University Veterinary Teaching Hospital, Gwangju, Republic of Korea; Instituto de Biotecnologia/UNAM

**Keywords:** endosomal acidification, PI3K/Akt, MEK/ERK, sapovirus, viral entry

## Abstract

Sapovirus, an important cause of acute gastroenteritis in humans and animals, travels from the early to the late endosomes and requires late endosomal acidification for viral uncoating. However, the signaling pathways responsible for these viral entry processes remain unknown. Here we demonstrate the receptor-mediated early activation of phosphatidylinositol 3-kinase (PI3K)/Akt and mitogen-activated protein extracellular signal-regulated kinase/extracellular signal-regulated kinase (MEK/ERK) signaling pathways involved in sapovirus entry processes. Both signaling pathways were activated during the early stage of porcine sapovirus (PSaV) infection. However, depletion of the cell surface carbohydrate receptors by pretreatment with sodium periodate or neuraminidase reduced the PSaV-induced early activation of these signaling pathways, indicating that PSaV binding to the cell surface carbohydrate receptors triggered these cascades. Addition of bile acid, known to be essential for PSaV escape from late endosomes, was also found to exert a stiffening effect to stimulate both pathways. Inhibition of these signaling pathways by use of inhibitors specific for PI3K or MEK or small interfering RNAs (siRNAs) against PI3K or MEK resulted in entrapment of PSaV particles in early endosomes and prevented their trafficking to late endosomes. Moreover, phosphorylated PI3K and ERK coimmunoprecipitated subunit E of the V-ATPase proton pump that is important for endosomal acidification. Based on our data, we conclude that receptor binding of PSaV activates both PI3K/Akt and MEK/ERK signaling pathways, which in turn promote PSaV trafficking from early to late endosomes and acidification of late endosomes for PSaV uncoating. These signaling cascades may provide a target for potent therapeutics against infections by PSaV and other caliciviruses.

**IMPORTANCE** Sapoviruses cause acute gastroenteritis in both humans and animals. However, the host signaling pathway(s) that facilitates host cell entry by sapoviruses remains largely unknown. Here we demonstrate that porcine sapovirus (PSaV) activates both PI3K/Akt and MEK/ERK cascades at an early stage of infection. Removal of cell surface receptors decreased PSaV-induced early activation of both cascades. Moreover, blocking of PI3K/Akt and MEK/ERK cascades entrapped PSaV particles in early endosomes and prevented their trafficking to the late endosomes. PSaV-induced early activation of PI3K and ERK molecules further mediated V-ATPase-dependent late endosomal acidification for PSaV uncoating. This work unravels a new mechanism by which receptor-mediated early activation of both cascades may facilitate PSaV trafficking from early to late endosomes and late endosomal acidification for PSaV uncoating, which in turn can be a new target for treatment of sapovirus infection.

## Abstract (precis)

Sapoviruses cause acute gastroenteritis in both humans and animals. However, the host signaling pathway(s) that facilitates host cell entry by sapoviruses remains largely unknown. Here we demonstrate that porcine sapovirus (PSaV) activates both PI3K/Akt and MEK/ERK cascades at an early stage of infection. Removal of cell surface receptors decreased PSaV-induced early activation of both cascades. Moreover, blocking of PI3K/Akt and MEK/ERK cascades entrapped PSaV particles in early endosomes and prevented their trafficking to the late endosomes. PSaV-induced early activation of PI3K and ERK molecules further mediated V-ATPase-dependent late endosomal acidification for PSaV uncoating. This work unravels a new mechanism by which receptor-mediated early activation of both cascades may facilitate PSaV trafficking from early to late endosomes and late endosomal acidification for PSaV uncoating, which in turn can be a new target for treatment of sapovirus infection.

## INTRODUCTION

Under normal physiological conditions, many cell types utilize the phosphatidylinositol 3-kinase/protein kinase B (known as Akt) (PI3K/Akt) and/or mitogen-activated protein extracellular signal-regulated kinase/extracellular signal-regulated kinase (MEK/ERK) pathway for various cellular functions, such as cell growth, proliferation, differentiation, survival, and intracellular vesicle trafficking (1, 2). Since the coding capacity of viral genomes is limited, viruses have evolved various ways to usurp host cell machinery to maintain their propagation (3–9). In this regard, a variety of viruses hijack the PI3K/Akt and MEK/ERK signaling pathways to create a favorable environment for their needs, ranging from their entry to their assembly and release (3–9). Particularly at the early stage of virus infection, a wide range of RNA and DNA viruses have been reported to activate the PI3K/Akt and/or MEK/ERK signaling cascades to mediate virus internalization and/or endosomal sorting (6). Receptor-mediated virus internalization via activation of the PI3K/Akt signaling pathway has been reported for the infections caused by many viruses, such as hepatitis C virus (HCV) (10), exogenous avian leukosis virus (ALV) (11), African swine fever virus (ASFV) (12), vaccinia virus (13), herpes simplex virus type 1 (HSV-1) (14, 15), porcine reproductive and respiratory syndrome virus (PRRSV) (16), Ebola virus (17), and adeno-associated virus type 2 (AAV-2) (18). On closer investigation, the PI3K/Akt signaling pathway was found to be involved in transferring human rhinovirus serotype 2 and dengue virus from the early to late endosomes (19, 20). In addition, influenza A virus (IAV)- and rotavirus-induced early activation of these signaling molecules mediates V-ATPase-dependent endosomal acidification, which is required for fusion (21, 22).

Caliciviruses, members of the *Caliciviridae* family, are small (27–40 nm), nonenveloped viruses containing a positive-sense single-stranded RNA of approximately 7 to 8 kb (23). They are formally classified into the following five genera: *Norovirus*, *Sapovirus*, *Lagovirus*, *Vesivirus*, and *Nebovirus* (23). Sapoviruses, together with noroviruses, are the most common causes of severe acute viral gastroenteritis in humans and animals (24, 25). The genus *Sapovirus* is currently classified into five genogroups (GI to GV) based on the complete sequences of viral capsid genes. Genogroups I, II, IV, and V are known to infect humans, whereas genogroup III contains the porcine sapovirus (PSaV) (25–27). Within the genus *Sapovirus*, the PSaV Cowden strain was isolated first from primary porcine kidney cells (28) and subsequently from a porcine kidney cell line (LLC-PK cells) (29) in the presence of porcine intestinal contents or bile acids, specifically glycochenodeoxycholic acid (GCDCA), as a medium supplement for virus replication (30, 31). Therefore, the PSaV Cowden strain serves as a suitable model for studies on sapovirus pathogenesis and molecular mechanisms involved in its life cycle (32).

Recently, we demonstrated that PSaV is internalized by clathrin- and cholesterol-mediated endocytosis, with the requirement of dynamin II and actin rearrangement, and that its uncoating occurs in the acidified late endosomes, after it has moved from early endosomes via microtubules (33). Although the PI3K/Akt and MEK/ERK signaling pathways are known to be involved in the entry of some viruses, their roles during PSaV entry have remained elusive. Here we demonstrate that PSaV-induced early activation of PI3K/Akt and MEK/ERK pathways facilitates trafficking of PSaV from early to late endosomes as well as the acidification of late endosomes for PSaV uncoating. Moreover, the interaction of PSaV with the cell surface carbohydrate receptor may trigger the activation of these signaling cascades. Our results will be important for the future development of prophylactic and therapeutic treatments against infection by sapoviruses and possibly other caliciviruses, such as human noroviruses, that create major public health concerns (34, 35).

## RESULTS

### PSaV-induced early activation of PI3K/Akt and MEK/ERK signaling pathways.

**FIG 1:**
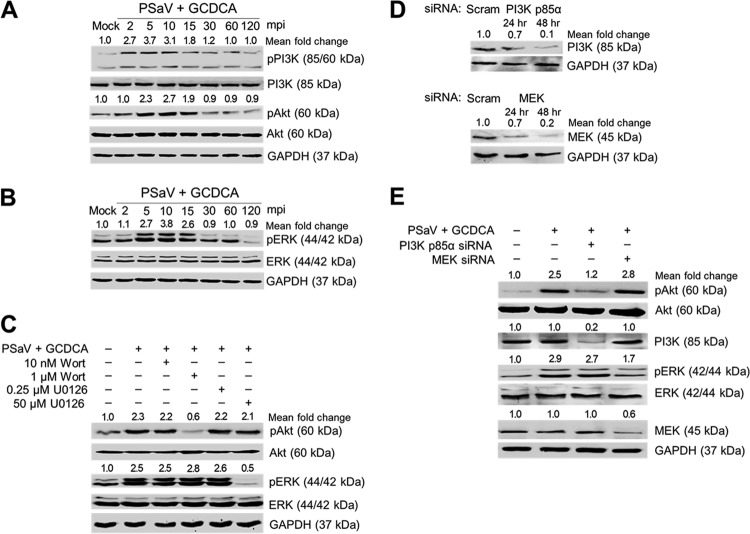
PSaV-induced early activation of PI3K/Akt and MEK/ERK signaling pathways. (A and B) LLC-PK cells were inoculated with the PSaV Cowden strain (MOI of 1 FFU/cell) in the presence of 200 μM GCDCA (bile acid) and then harvested at the indicated time points. The levels of PI3K, Akt, ERK, pPI3K p85 (Tyr458)/p55 (Tyr199), pAkt (Ser473), pERK (Thr202/Tyr204), and GAPDH were evaluated by Western blotting using specific antibodies against the target proteins. GAPDH was used as a loading control. (C) LLC-PK cells were mock pretreated or pretreated with wortmannin (PI3K inhibitor) or U0126 (MEK inhibitor) at the indicated doses for 1 h at 37°C and then infected with or without PSaV in the presence of 200 μM GCDCA. Cell lysates were harvested at 5 min postinoculation (mpi). The expression levels of pAkt (Ser473), Akt, pERK (Thr202/Tyr204), ERK, and GAPDH were evaluated by Western blotting. GAPDH was used as a loading control. (D) LLC-PK cells transfected with scrambled siRNA (Scram) or siRNA against PI3K p85α or MEK were harvested at 24 and 48 h posttransfection. The downregulation of each protein by siRNA knockdown was evaluated by Western blotting using antibodies specific for each protein. GAPDH was used as a loading control. (E) LLC-PK cells transfected with or without each siRNA were incubated with PSaV (MOI of 1 FFU/cell) in the presence of 200 μM GCDCA. Cell lysates were harvested at 5 mpi. The expression levels of pAkt (Ser473), PI3K, pERK (Thr202/Tyr204), MEK, and GAPDH were determined by Western blotting. GAPDH was used as a loading control. The intensity of each target protein relative to that of GAPDH was determined by densitometric analysis and is indicated above each lane.

**FIG 2:**
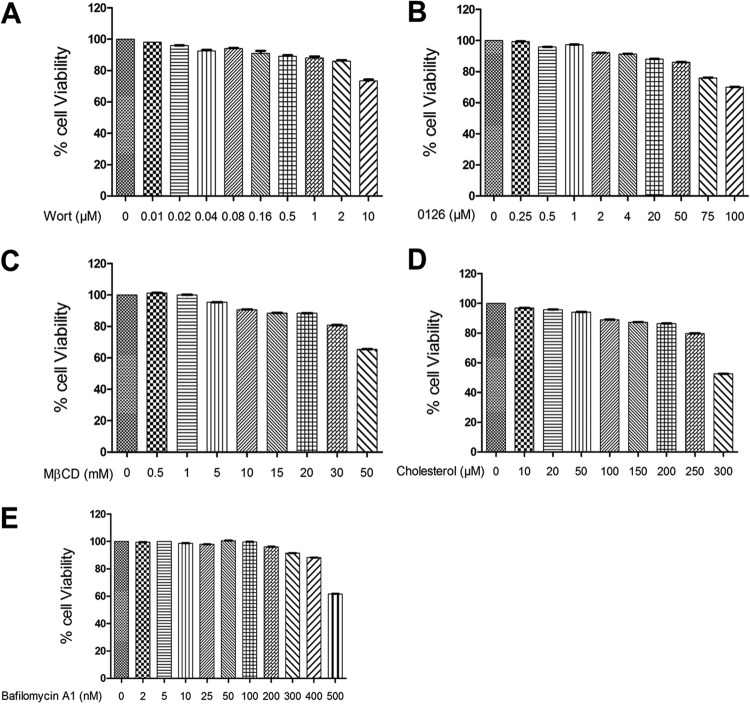
Determination of chemical-mediated cytotoxicity in LLC-PK cells by MTT assay. (A to E) LLC-PK cells grown in 96-well plates were incubated with various concentrations of the indicated chemicals in triplicate for 24 h at 37°C. Afterward, the chemical-containing medium was thoroughly removed and replaced with 200 μl of MTT solution for 4 h at 37°C. Each well was incubated with 100 μl of DMSO for 10 min at room temperature. Cell viability was measured using an enzyme-linked immunosorbent assay (ELISA) reader to obtain the optical density at 570 nm.

To examine whether PSaV could activate PI3K/Akt and MEK/ERK signaling pathways during the early stage of the viral life cycle, LLC-PK cells were infected with or without PSaV strain Cowden (multiplicity of infection [MOI] of 1) in the presence of 200 μM GCDCA for the times indicated in the figures. Using antibodies specific for PI3K, phosphorylated PI3K (pPI3K), Akt, phosphorylated Akt (pAkt), ERK, and phosphorylated ERK (pERK), Western blotting was performed with cell lysates prepared after the above treatments. Compared to that in mock-inoculated cells, activation of pPI3K, pAkt, and pERK was observed in virus-infected cells as early as 2 min postinfection (mpi) for pPI3K and 5 mpi for pAkt and pERK, was sustained at a high level until 15 min, and then declined thereafter (Fig. 1A and B). To check whether the phosphorylation of Akt or ERK could be regulated by the upstream molecules PI3K and MEK, cells were pretreated with noncytotoxic concentrations of inhibitors specific for PI3K (wortmannin) or MEK (U0126) (Fig. 2). As shown in Fig. 1C, each inhibitor specifically and efficiently inhibited only the corresponding downstream molecule. Furthermore, knockdown of PI3K p85α or MEK by transfection with specific small interfering RNAs (siRNAs) (Fig. 1D) resulted in a significant reduction of pAkt or pERK, respectively (Fig. 1E). These results suggested that PSaV infection concomitantly and independently induces early activation of PI3K/Akt and MEK/ERK signaling pathways.

### Activation of PI3K/Akt and MEK/ERK signaling pathways by PSaV and bile acid.

**FIG 3:**
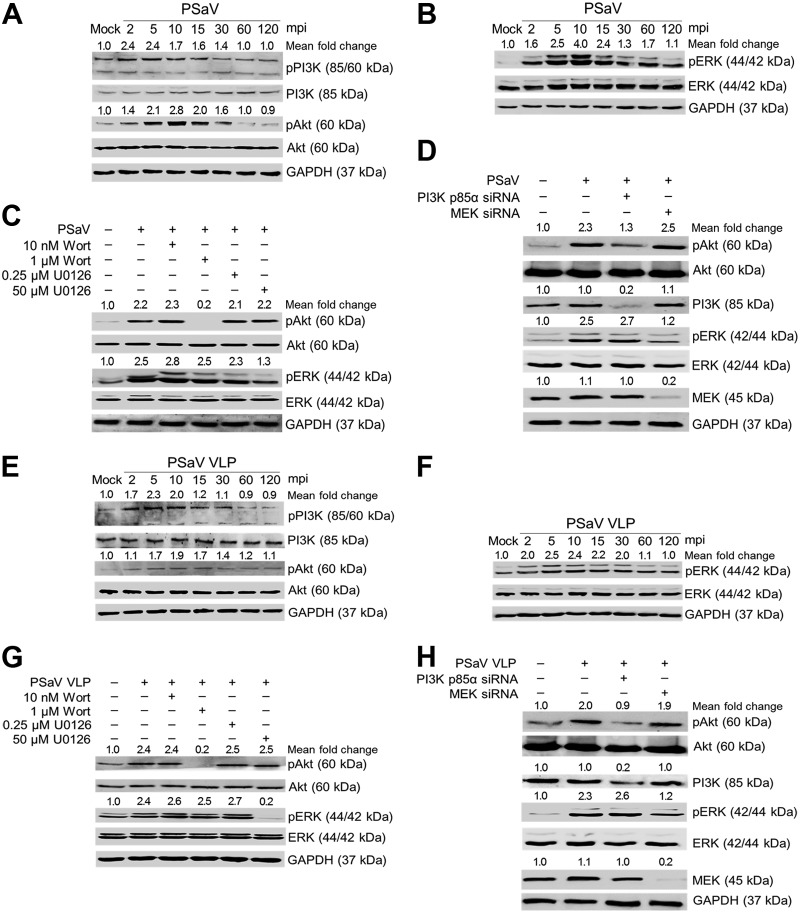
Activation of PI3K/Akt and MEK/ERK signaling pathways by direct interaction of PSaV in the absence of GCDCA. (A and B) LLC-PK cells were incubated with PSaV (MOI of 1 FFU/cell) in the absence of GCDCA (bile acid) and then harvested at the indicated time points. The levels of PI3K, Akt, ERK, pPI3K p85 (Tyr458)/p55 (Tyr199), pAkt (Ser473), pERK (Thr202/Tyr204), and GAPDH were evaluated by Western blotting using specific antibodies against the target proteins. GAPDH was used as a loading control. (C and D) LLC-PK cells were mock pretreated or pretreated with wortmannin (PI3K inhibitor) or U0126 (MEK inhibitor) at the indicated doses for 1 h at 37°C (C) or transfected with or without siRNAs against PI3K p85α or MEK (D) and then infected with or without PSaV in the absence of GCDCA. Cell lysates were harvested at 5 mpi. The expression levels of pAkt (Ser473), Akt, pERK (Thr202/Tyr204), ERK, and GAPDH were evaluated by Western blotting. GAPDH was used as a loading control. (E and F) LLC-PK cells were incubated with PSaV VLPs (10 μg/ml), and the cell lysates were harvested at 5 mpi and prepared for Western blotting as described above. (G and H) LLC-PK cells were mock pretreated or pretreated with wortmannin or U0126 at the indicated doses for 1 h at 37°C (G) or transfected with or without siRNAs against PI3K p85α or MEK (H) and then incubated with or without PSaV VLPs. Cell lysates were harvested at 5 mpi. The expression levels of pAkt (Ser473), Akt, pERK (Thr202/Tyr204), ERK, and GAPDH were evaluated by Western blotting. GAPDH was used as a loading control. The intensity of each target protein relative to that of GAPDH was determined by densitometric analysis and is indicated above each lane.

It is well known that the PSaV Cowden strain can replicate *in vitro* in the presence of bile acid (31). To test whether addition of infectious PSaV virions to cells in the absence of bile acids could induce early activation of both signaling pathways, LLC-PK cells were infected with or without PSaV at an MOI of 1 in the absence of any bile acid for the times indicated in the figures. The results showed that PSaV induced phosphorylation of PI3K, Akt, and ERK as early as 2 mpi, and this became clearly obvious at 5 mpi (Fig. 3A and B). In addition, pretreatment of cells with the specific inhibitors wortmannin and U0126 and transfection with siRNAs against PI3K p85α and MEK abolished phosphorylation of the downstream effectors, Akt and ERK, respectively (Fig. 3C and D).

**FIG 4:**
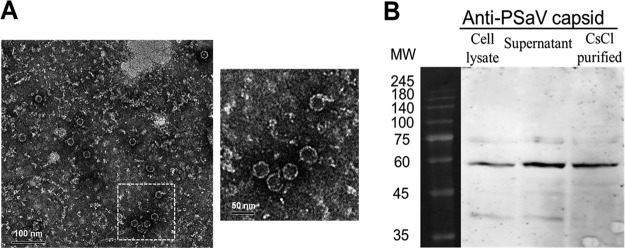
Electron micrograph and Western blot analyses of PSaV Cowden strain VLPs. (A) PSaV VLPs harvested from recombinant baculovirus-infected Sf9 cells at 72 h postinfection were purified by CsCl density gradient ultracentrifugation and visualized by negative staining with 3% phosphotungstic acid (pH 7.0) under an electron microscope. The inset shows a higher magnification of the left panel. (B) Cell lysates of recombinant baculovirus-infected Sf9 cells at 72 h postinfection, their supernatants, and CsCl-purified VLPs were separated by sodium dodecyl sulfate-polyacrylamide gel electrophoresis, and VLPs were detected by Western blot analysis using anti-PSaV capsid hyperimmune antisera.

To further confirm the above results, PSaV Cowden strain virus-like particles (VLPs) were employed in the absence of bile acid. The VLPs produced from Spodoptera frugiperda ovarian (Sf9) cells had a size of 35 to 40 nm and appeared empty by electron microscopy (EM) due to the lack of viral nucleic acids (Fig. 4). Western blotting with anti-PSaV capsid hyperimmune antisera detected a 58-kDa protein, as expected (Fig. 4) and consistent with previous reports on other caliciviruses (36–38). Addition of PSaV VLPs (10 μg/ml) for the indicated times induced the early activation of PI3K, Akt, and ERK (Fig. 3E and F); similarly, inhibitory effects were observed on these PSaV VLP-induced signaling pathways upon treatment with wortmannin and U0126 and upon transfection with siRNAs against PI3K p85α or MEK (Fig. 3G and H). These results indicated that PSaV infection induced early activation of PI3K/Akt and MEK/ERK signaling pathways in the absence of bile acids, which is known to be essential for PSaV replication.

**FIG 5:**
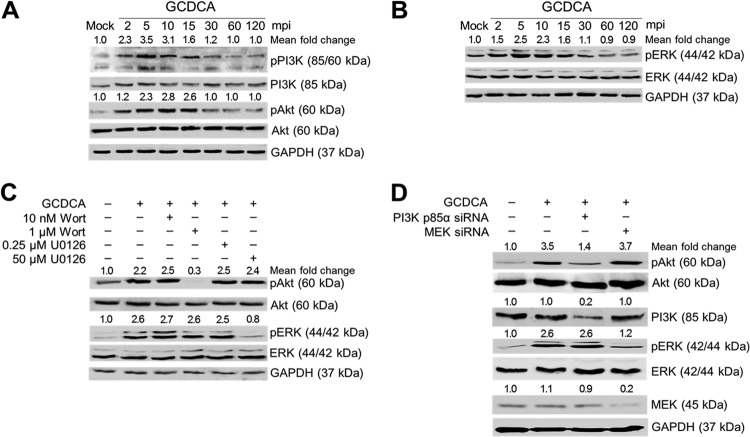
Bile acid-induced early activation of PI3K/Akt and MEK/ERK signaling pathways. (A and B) LLC-PK cells were treated with or without 200 μM GCDCA and then harvested at the indicated time points. The levels of PI3K, Akt, ERK, pPI3K p85 (Tyr458)/p55 (Tyr199), pAkt (Ser473), pERK (Thr202/Tyr204), and GAPDH were determined by Western blotting using specific antibodies against the target proteins. GAPDH was used as a loading control. (C) LLC-PK cells were mock pretreated or pretreated with wortmannin (PI3K inhibitor) or U0126 (MEK inhibitor) at the indicated doses for 1 h at 37°C and then treated with or without 200 μM GCDCA. Cell lysates were harvested at 5 min posttreatment. The expression levels of pAkt (Ser473), Akt, pERK (Thr202/Tyr204), ERK, and GAPDH were evaluated by Western blotting. GAPDH was used as a loading control. (D) LLC-PK cells were transfected with or without siRNAs against PI3K p85α or MEK and then treated with or without 200 μM GCDCA. Cell lysates were harvested at 5 min posttreatment. The expression levels of pAkt (Ser473), PI3K, pERK (Thr202/Tyr204), MEK, and GAPDH were evaluated by Western blotting. GAPDH was used as a loading control. The intensity of each target protein relative to that of GAPDH was determined by densitometric analysis and is indicated above each lane.

A previous report demonstrated that bile acids play a critical role in viral genome release from late endosomes into the cytoplasm to initiate viral replication (39). The PI3K/Akt and MEK/ERK signaling pathways can be activated through the G-protein-coupled receptor (40, 41), which has been identified as a bile acid-activated membrane receptor (42). Hence, we next examined whether bile acids could activate PI3K/Akt and MEK/ERK signaling pathways. LLC-PK cells were treated with the conjugated bile acid GCDCA for the times indicated in the figures. Western blotting showed that addition of GCDCA to the cells activated PI3K/Akt and MEK/ERK signaling pathways as early as 2 min after the addition (Fig. 5A and B). Moreover, the specific inhibitors of PI3K (wortmannin) and MEK (U0126) and siRNA-mediated knockdown of PI3K and MEK significantly blocked activation of the downstream molecules, Akt and ERK (Fig. 5C and D). Taking these results together, it appears that PSaV infection can activate both PI3K/Akt and MEK/ERK signaling pathways by itself and that bile acids may enhance these effects.

### Receptor-mediated activation of PI3K/Akt and MEK/ERK signaling pathways.

**FIG 6:**
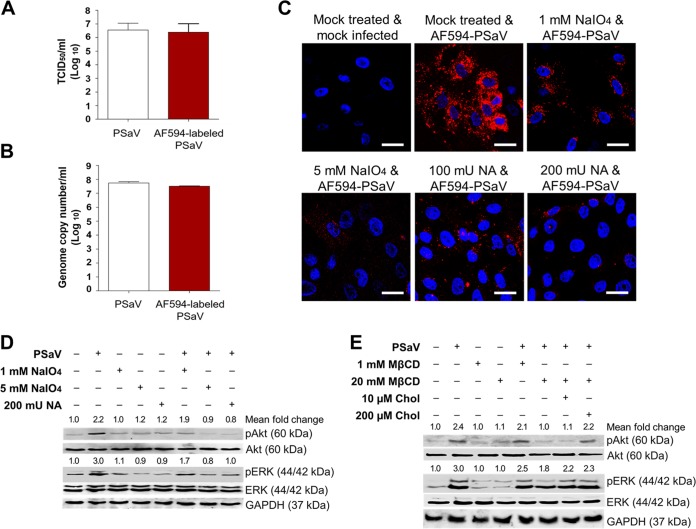
Activation of PI3K/Akt and MEK/ERK signaling pathways by interaction of PSaV with cell surface carbohydrate receptors. (A and B) LLC-PK cells were infected with mock-labeled PSaV particles or Alexa Fluor 594 (AF594)-labeled PSaV particles and incubated for 36 h at 37°C in the presence of 200 μM GCDCA. The cells were then harvested by freezing and thawing, and the virus titers were determined by TCID_50_ assay (A) and the genome copy number by real-time RT-PCR (B) as described in Materials and Methods. (C) LLC-PK cells were treated with or without 1 or 5 mM sodium periodate (NaIO_4_) or 100 or 200 mU Vibrio cholerae neuraminidase (NA) to remove carbohydrates or sialic acids from the cell surface, respectively, incubated with AF594-labeled PSaV particles (approximately 415 particles per cell) for 30 min at 4°C in the absence of 200 μM GCDCA, and subsequently examined by confocal microscopy. This experiment was repeated three independent times, and one representative set of results is shown. Bars, 20 μm. (D and E) LLC-PK cells were treated with or without 1 or 5 mM NaIO_4_ or 200 mU NA for 1 h at 37°C (D) or treated with 1 or 20 mM MβCD for 1 h at 37°C or with 10 or 200 µM soluble cholesterol for 30 min at 37°C to examine the effect of cholesterol replenishment following MβCD-mediated depletion (E), followed by infection with PSaV (MOI of 1 FFU/cell) in the presence of 200 μM GCDCA. Cell lysates were harvested at 5 mpi. Antibodies against pAkt (Ser473), Akt, pERK (Thr202/Tyr204), ERK, and GAPDH were used to evaluate the expression level of each target protein by Western blotting. GAPDH was used as a loading control. The intensity of each target protein relative to that of GAPDH was determined by densitometric analysis and is indicated above each lane.

PSaV-induced early activation of PI3K/Akt and MEK/ERK signaling pathways may be mediated by the interaction between PSaV and its cell surface receptors. Previously, we demonstrated that both α2,3- and α2,6-linked sialic acids on *O*-linked glycoproteins act as cell surface attachment receptors (43). Here we examined whether removal of cell surface carbohydrates, including both α2,3- and α2,6-linked sialic acids, inhibits PI3K/Akt and MEK/ERK signaling pathways. We first examined the effect of removal of α2,3- and α2,6-linked sialic acids on Alexa Fluor 594 (AF594)-labeled PSaV binding. Labeling of PSaV with AF594 could not affect its infectivity in comparison to that of mock-labeled PSaV (Fig. 6A and B). Therefore, LLC-PK cells were pretreated with or without 1 or 5 mM sodium periodate (NaIO_4_), which is known to remove carbohydrate groups without altering proteins or membranes; cells were also pretreated with or without 100 or 200 mU Vibrio cholerae neuraminidase (NA), which cleaves α2,3-linked, α2,6-linked, and α2,8-linked sialic acids from the underlying glycans (43). In comparison to that with the mock-treated cells, pretreatment with NaIO_4_ and NA inhibited binding of AF594-labeled PSaV to the cells (Fig. 6C), confirming the previous results (43). Removal of carbohydrate moieties from the mock-inoculated cells by use of 1 or 5 mM NaIO_4_ or of sialic acids by use of NA had no effects on the activation of PI3K/Akt and MEK/ERK signaling pathways (Fig. 6D). This indicated that pretreatment with NaIO_4_ and NA did not alter both signaling pathways. However, inhibition of PSaV binding to cell surface receptors by pretreatment with NaIO_4_ or NA reduced the activation of PI3K/Akt and MEK/ERK signaling pathways (Fig. 5D). These data suggested that early activation of PI3K/Akt and MEK/ERK signaling pathways could be induced by the interaction between PSaV and its cell surface attachment receptors.

### Involvement of PI3K/Akt and MEK/ERK signaling pathways in PSaV trafficking.

The above-described results imply that PSaV-induced early activation of PI3K/Akt and MEK/ERK signaling pathways might be involved in the virus entry process. In our previous report, we demonstrated that PSaV enters cells through clathrin- and cholesterol-mediated endocytosis and travels from early to late endosomes (33). Since these membrane lipid rafts compartmentalize cellular processes by acting as a signaling platform (44), we tested whether cholesterol-perturbing drugs could affect these signaling pathways in response to PSaV infection. Interestingly, pretreatment of cells with the cholesterol-perturbing drug methyl-beta-cyclodextrin (MβCD) reduced the activation of PI3K/Akt and MEK/ERK signaling pathways in a dose-dependent manner (Fig. 6E), and this inhibitory effect was restored by the addition of soluble cholesterol (Fig. 6E). These results suggested that PSaV entry via cholesterol-mediated endocytosis might activate both signaling pathways and vice versa.

**FIG 7:**
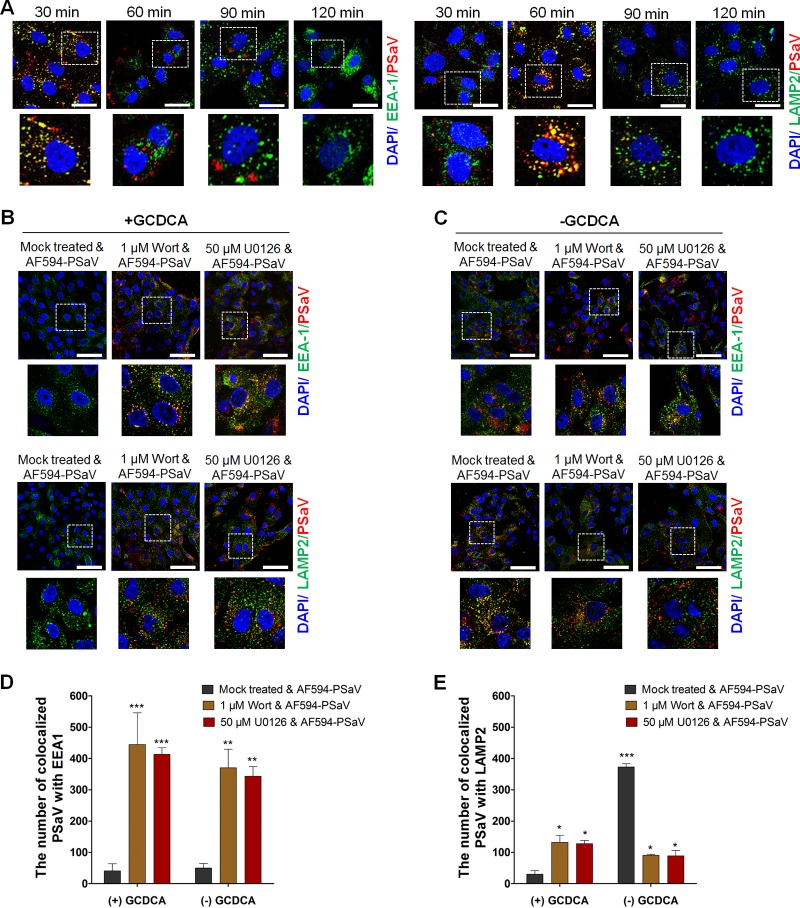
Inhibition of PSaV trafficking by blockade of PI3K/Akt and MEK/ERK signaling pathways. (A) LLC-PK cells were incubated with Alexa Fluor 594 (AF594)-labeled PSaV particles (approximately 415 particles per cell) for the indicated times at 37°C in the presence of 200 μM GCDCA. The cells were then fixed, permeabilized, and further incubated with a monoclonal antibody against the early endosome marker EEA1 or the late endosome marker LAMP2. After incubation with a FITC-conjugated anti-mouse IgG antibody, the cells were processed for confocal microscopy to determine the colocalization of AF594-labeled PSaV particles with the early endosome marker EEA1 or the late endosome marker LAMP2. The boxed areas are magnified and shown under each panel. (B and C) LLC-PK cells were pretreated with or without wortmannin (PI3K inhibitor) or U0126 (MEK inhibitor) for 1 h at 37°C and then infected with AF594-labeled PSaV particles (approximately 415 particles per cell) in the presence (B) or absence (C) of 200 μM GCDCA for 3 h. After fixation and permeabilization, the cells were incubated with a monoclonal antibody against EEA1 or LAMP2 and then with a FITC-conjugated secondary antibody to visualize colocalization of AF594-labeled PSaV particles with EEA1 or LAMP2. The boxed areas are magnified and shown under each panel. All experiments were performed in triplicate, and a representative set of results is shown. Bars, 10 μm (A) and 20 μm (B and C). (D and E) Quantification of AF595-labeled PSaV particles colocalized with the early endosome marker EEA1 (D) and the late endosome marker LAMP2 (E) was performed using 10 confocal microscopy images of cells treated under the conditions described above by use of the ImageJ program. Quantification of signals was made with a threshold of 0.03 to 1.3 µm^2^ as described in Materials and Methods.

We next examined whether both signaling pathways could be involved in PSaV trafficking from early to late endosomes in the presence or absence of 200 μM GCDCA. Before evaluating the involvement of both signaling pathways in PSaV entry, we first examined endosomal trafficking of the PSaV Cowden strain. Colocalization of AF594-labeled PSaV particles with an early endosome marker (early endosome antigen 1 [EEA1]) gradually increased until 30 mpi and decreased thereafter (Fig. 7A), whereas AF594-labeled PSaV particles were colocalized with a late endosome marker (lysosome-associated membrane protein 2 [LAMP2]) until 60 mpi and its interaction decreased thereafter (Fig. 7A). Confirming the results of our recent report (33), these data indicated that uncoating of PSaV particles was completed at 90 mpi.

The bile acid GCDCA plays a critical role in PSaV uncoating from late endosomes (39). Therefore, the colocalization of AF594-labeled PSaV particles with EEA1 or LAMP2 disappeared in the cytoplasm in the presence of GCDCA after 3 h postinoculation (Fig. 7B and D). In the absence of GCDCA, AF594-labeled PSaV particles were colocalized with LAMP2 even after 3 h postinoculation (Fig. 7C and E) (39). We next examined whether PSaV-induced early activation of PI3K/Akt and MEK/ERK signaling pathways is involved in PSaV trafficking from early to late endosomes. Pretreatment of cells with specific inhibitors of PI3K (wortmannin) and MEK (U0126) trapped most of the AF594-labeled PSaV particles in the early endosome, regardless of the addition of GCDCA to the medium (Fig. 7B to E), even when cells were incubated for 3 h postinoculation.

**FIG 8:**
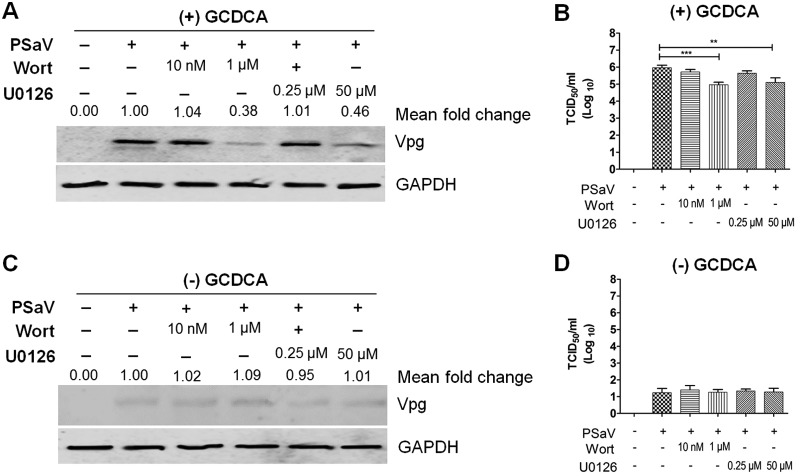
Inhibition of the PI3K/Akt and MEK/ERK signaling pathways affects PSaV infectivity and viral protein expression. (A to D) LLC-PK cells were pretreated with noncytotoxic concentrations of wortmannin or U0126 for 1 h at 37°C and then infected with PSaV (MOI of 1 FFU/cell) for 36 h in the presence (A and B) or absence (C and D) of 200 μM GCDCA. (A and C) Levels of PSaV VPg protein were determined by Western blotting. GAPDH was used as a loading control. The intensity of VPg relative to that of GAPDH was determined by densitometric analysis and is indicated above each lane. (B and D) Viral titers were determined by TCID_50_ assay. The data are presented as means and standard deviations of the results of three independent experiments. Differences were evaluated using one-way analysis of variance. *, *P* < 0.05; **, *P* < 0.01; ***, *P* < 0.001; ****, *P* < 0.00001.

**FIG 9:**
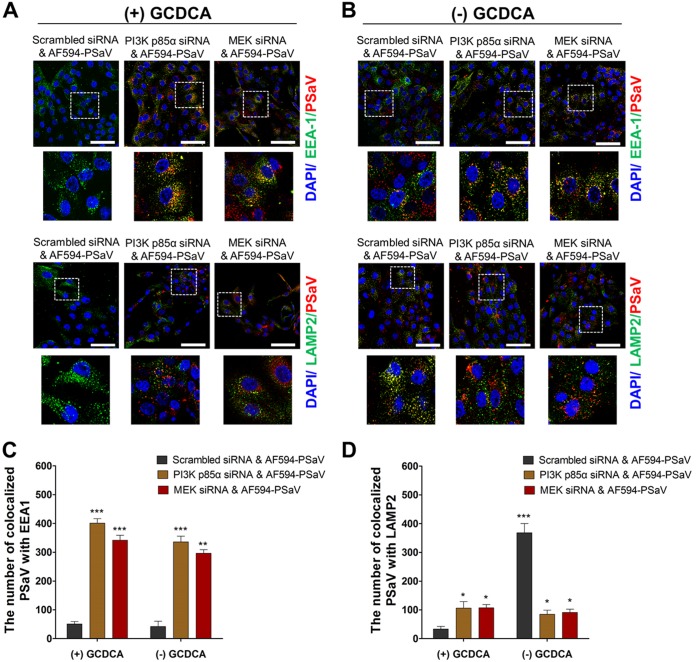
Silencing of PI3K and MEK traps PSaV particles in early endosomes. (A and B) LLC-PK cells were transfected with scrambled siRNA or siRNA against PI3K p85α or MEK and then incubated with Alexa Fluor 594 (AF594)-labeled PSaV particles (approximately 415 particles per cell) for 3 h in the presence (A) or absence (B) of 200 μM GCDCA. After fixation and permeabilization, the cells were incubated with a monoclonal antibody against the early endosome marker EEA1 or the late endosome marker LAMP2 and then with a FITC-conjugated secondary antibody and processed for confocal microscopy to determine the colocalization of AF594-labeled PSaV particles with EEA1 or LAMP2. The boxed areas are magnified and shown under each panel. All experiments were performed in triplicate, and a representative set of results is shown. Bars, 20 μm. (C and D) Quantification of AF595-labeled PSaV particles colocalized with the early endosome marker EEA1 (C) and the late endosome marker LAMP2 (D) was performed using 10 confocal microscopy images of cells treated under the conditions described above by use of the ImageJ program. Quantification of signals was made with a threshold of 0.03 to 1.3 µm^2^ as described in Materials and Methods.

Activation and manipulation of PI3K/Akt and MEK/ERK signaling pathways by the PSaV trafficking process might finally affect the virus infectivity. To check whether inhibition of both signaling pathways could influence PSaV infectivity, LLC-PK cells were first pretreated with wortmannin or U0126 for 1 h, followed by PSaV infection (MOI = 1 focus-forming unit [FFU]/cell) in the presence or absence of 200 μM GCDCA. In the presence of GCDCA, inhibition of both PI3K/Akt and MEK/ERK pathways reduced PSaV VPg expression (Fig. 8A), as well as the viral progeny (Fig. 8B), in a dose-dependent manner. However, the PSaV Cowden strain could not replicate properly in the absence of GCDCA (Fig. 8A and B) (28, 29), regardless of pretreatment with inhibitors (Fig. 8C and D). We further confirmed these results by checking the effect of PI3K p85α- or MEK-specific siRNAs on colocalization of PSaV particles with EEA1 and LAMP2. Regardless of the addition of GCDCA, siRNA knockdown of PI3K p85α or MEK trapped the PSaV particles in early endosomes, even when cells were incubated for 3 h postinoculation (Fig. 9). Taken together, these data suggested that both PI3K/Akt and MEK/ERK signaling pathways could be involved in PSaV trafficking from early to late endosomes.

### PSaV-induced pPI3K and pERK bind to V-ATPase.

**FIG 10:**
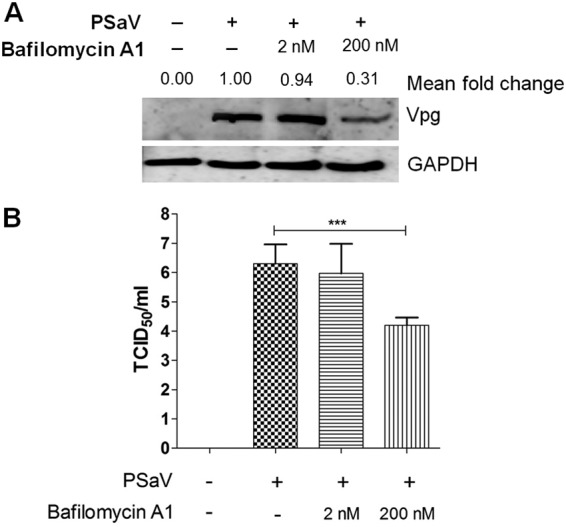
V-ATPase-induced endosomal acidification is required for PSaV infection. (A and B) LLC-PK cells were pretreated with or without noncytotoxic concentrations of bafilomycin A1 for 1 h at 37°C and then infected with or without PSaV (MOI of 1 FFU/cell) for 36 h in the presence of 200 μM GCDCA. (A) Levels of PSaV VPg protein were determined by Western blotting. GAPDH was used as a loading control. The intensity of VPg relative to that of GAPDH was determined by densitometric analysis and is indicated above each lane. (B) Viral titers were determined by TCID_50_ assay. All experiments were performed in triplicate, and a representative set of results is shown. The data are presented as means and standard deviations of the means of the results of three independent experiments. Differences were evaluated using one-way analysis of variance. *, *P* < 0.05; **, *P* < 0.01; ***, *P* < 0.001; ****, *P* < 0.00001.

**FIG 11:**
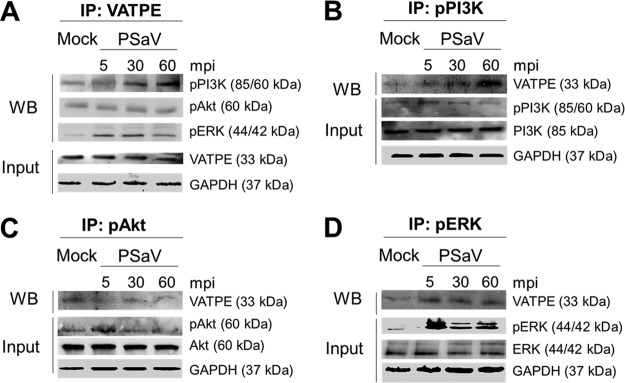
Coimmunoprecipitation (IP) of phosphorylated PI3K and ERK signaling molecules with the V-ATPase pump. (A to D) Serum-starved LLC-PK cells were inoculated with or without the PSaV Cowden strain (MOI of 1 FFU/cell) for the indicated times. Subsequently, the cell lysates were immunoprecipitated using antibodies specific for V_1_ subunit E of V-ATPase (A), pPI3K (B), pAkt (C), and pERK (D). The coimmunoprecipitated products were analyzed by Western blotting to detect pPI3K p85 (Tyr458)/p55 (Tyr199), pAkt (Ser473), pERK (Thr202/Tyr204), and V_1_ subunit E by use of the relevant antibodies. GAPDH was used as a loading control.

**FIG 12:**
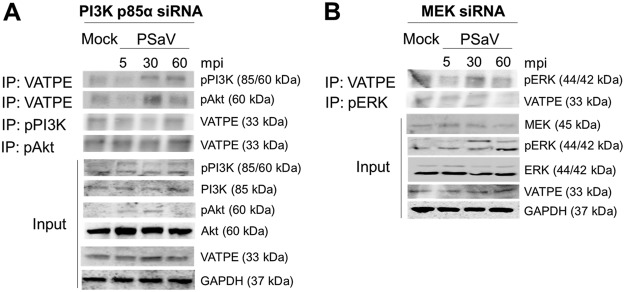
Silencing of PI3K and MEK inhibits the coimmunoprecipitation of phosphorylated PI3K and ERK signaling molecules with the V-ATPase pump. (A and B) LLC-PK cells were transfected with scrambled siRNA or siRNA against PI3K p85α (A) or MEK (B) and then incubated with the PSaV Cowden strain (MOI of 1 FFU/cell) in the presence of 200 μM GCDCA for the indicated times. Subsequently, the cell lysates were immunoprecipitated using antibodies specific for V_1_ subunit E of V-ATPase, pPI3K, pAkt, and pERK. The coimmunoprecipitated products were analyzed by Western blotting to detect pPI3K p85 (Tyr458)/p55 (Tyr199), pAkt (Ser473), pERK (Thr202/Tyr204), and V_1_ subunit E by use of the relevant antibodies. GAPDH was used as a loading control.

**FIG 13:**
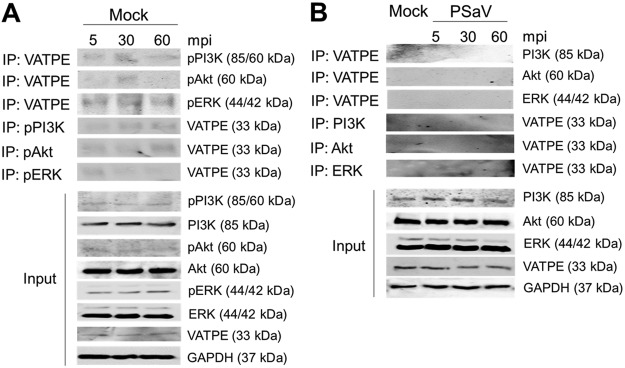
V-ATPase cannot immunoprecipitate phosphorylated PI3K, Akt, and ERK in mock-infected cells or nonphosphorylated PI3K, Akt, and ERK in PSaV-infected cells. (A) LLC-PK cells were mock infected for the indicated times. Subsequently, the cell lysates were immunoprecipitated using antibodies specific for V_1_ subunit E of V-ATPase, pPI3K, pAkt, and pERK. The coimmunoprecipitated products were analyzed by Western blotting to detect pPI3K p85 (Tyr458)/p55 (Tyr199), pAkt (Ser473), pERK (Thr202/Tyr204), and V_1_ subunit E by use of the relevant antibodies. GAPDH was used as a loading control. (B) LLC-PK cells were incubated with or without the PSaV Cowden strain (MOI of 1 FFU/cell) in the presence of 200 μM GCDCA for the indicated times. Subsequently, the cell lysates were immunoprecipitated using antibodies specific for V_1_ subunit E of V-ATPase, PI3K, Akt, and ERK. The coimmunoprecipitated products were analyzed by Western blotting to detect PI3K, Akt, ERK, and V_1_ subunit E by use of the relevant antibodies. GAPDH was used as a loading control.

In a previous report (45), PSaV infection induced late endosomal acidification, which is required for viral uncoating and genome release into the cytoplasm. Influenza A virus- and rotavirus-induced early activation of PI3K and ERK signaling molecules is known to mediate V-ATPase-dependent endosomal acidification, which is required for viral uncoating (21, 22). Hence, we first examined the need for acidification, especially through the activation of V-ATPase, by treatment with the V-ATPase inhibitor bafilomycin A1. Compared to that in mock-treated and PSaV-infected cells, pretreatment of bafilomycin A1 reduced PSaV VPg expression (Fig. 10A), as well as the viral progeny (Fig. 10B), in a dose-dependent manner, indicating that the role of V-ATPase-dependent endosomal acidification is PSaV uncoating. We next investigated whether the phosphorylated PI3K, Akt, and ERK could mediate endosomal acidification through direct interaction with V-ATPase upon PSaV infection. To assess this, LLC-PK cells were either mock infected or infected with the PSaV Cowden strain for the indicated time points, and the cell lysates were immunoprecipitated with antibodies specific for subunit E of the V_1_ domain of the V-ATPase, pPI3K, pAkt, or pERK. The results showed that the antibody specific for subunit E of the V_1_ domain of the V-ATPase precipitated pPI3K and pERK from the infected cell lysate (Fig. 11A). Likewise, antibodies specific for pPI3K or pERK coimmunoprecipitated the V_1_ domain of the V-ATPase from the infected cell lysate (Fig. 11B and D). However, subunit E of the V_1_ domain of the V-ATPase had no significant effect on the immunoprecipitation of pAkt from the infected cell lysate (Fig. 11A), and vice versa (Fig. 11C). Furthermore, transfection of siRNAs against PI3K p85α or MEK inhibited the coimmunoprecipitation of V-ATPase with pPI3K and pERK and vice versa (Fig. 12), supporting the above results. As a control, the cell lysates of mock-infected cells harvested at the same time points were immunoprecipitated with antibodies specific for subunit E of the V_1_ domain of the V-ATPase, pPI3K, pAkt, or pERK. The results showed that all three molecules (pPI3K, pAkt, and pERK) did not significantly coimmunoprecipitate V-ATPase and vice versa (Fig. 13). To confirm that V-ATPase is not coimmunoprecipitated with nonphosphorylated PI3K, Akt, or ERK, the lysates of LLC-PK cells infected with or without the PSaV Cowden strain were immunoprecipitated with an antibody specific for subunit E of the V_1_ domain of the V-ATPase or with antibodies specific for nonphosphorylated PI3K, Akt, or ERK, and then coimmunoprecipitation of each target protein was determined by Western blot analysis. The results showed that antibody against nonphosphorylated PI3K, Akt, or ERK did not coimmunoprecipitate V-ATPase and vice versa (Fig. 13). These results suggested that pPI3K and pERK molecules, which were activated in the early stage of infection by PSaV, could interact with V-ATPase for the late endosomal acidification for PSaV uncoating.

**FIG 14:**
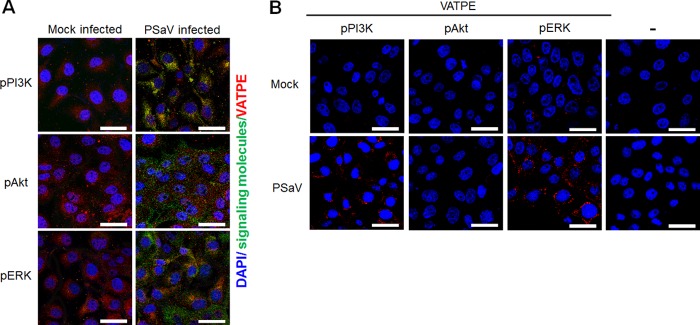
Direct interaction of pPI3K and pERK with subunit E of the V-ATPase V_1_ domain as determined by immunofluorescence assay and the Duolink proximity ligation assay. (A) Serum-starved LLC-PK cells were either mock incubated or incubated with the PSaV Cowden strain (MOI = 1 FFU/cell) in the presence of 200 μM GCDCA. Subsequently, the cells were fixed, permeabilized, and incubated with a mixture of primary mouse anti-V-ATPase E subunit and rabbit anti-pPI3K, -pAkt, or -pERK antibody overnight at 4°C. The cells were then incubated with irrelevant secondary antibodies for 1 h at room temperature and processed for confocal microscopy. (B) Serum-starved LLC-PK cells were either mock incubated or incubated with the PSaV Cowden strain (MOI = 1 FFU/cell) in the presence of 200 μM GCDCA. Subsequently, the cells were fixed, permeabilized, and incubated with or without a mixture of primary mouse anti-V-ATPase E subunit and rabbit anti-pPI3K, -pAkt, or -pERK antibody overnight at 4°C. The Duolink PLA was performed as described in Materials and Methods, and the signals are represented by red dots. Representative images are shown. Bars, 20 μm.

To corroborate the above immunoprecipitation results, the colocalization of subunit E of the V_1_ domain of the V-ATPase with pPI3K, pAkt, and pERK in PSaV-infected cells was further evaluated by confocal microscopy. Compared to those in mock-infected cells, pPI3K and pERK, but not pAkt, were colocalized with V-ATPase in PSaV-infected cells (Fig. 14A). We further confirmed these results by using the Duolink proximity ligation assay (PLA). In this assay, the signal from the interaction of two proteins in close proximity (40 nm or less) is easily visible as a distinct fluorescent spot (22). The assay was able to identify subunit E of the V_1_ domain of V-ATPase with pPI3K or pERK as a partner in LLC-PK cells infected with the PSaV Cowden strain, as indicated by the red dots (Fig. 14B).

## DISCUSSION

Many viruses not only pirate host cell machinery but also induce a variety of host cell signaling pathways that modulate the host intracellular environment at different stages of the life cycle to promote the virus life cycle (46). For instance, PSaV hijacks the host cellular translation initiation machinery by recruiting the eIF4F complex through a direct eIF4E interaction with PSaV VPg (32). There is accumulating evidence that PI3K/Akt and/or MEK/ERK signaling pathways might be hijacked by a wide variety of viruses, including either DNA or RNA viruses. These pathways are used to exhibit various supportive functions at several steps of the viral replication cycle, such as facilitating various steps of viral entry, augmenting viral replication, and promoting viral assembly and release (1, 2, 5, 6). Here we demonstrate that receptor-mediated activation of PI3K/Akt and MEK/ERK signaling pathways by the PSaV Cowden strain facilitates viral trafficking from early to late endosomes and promotes the V-ATPase-dependent late endosomal acidification that is required for PSaV uncoating.

To overcome and bypass the membrane and cytosolic barriers during viral entry, many viruses commonly use endocytic pathways to enter cells, and incoming viruses thus reach the endosomal compartments, where the uncoating process takes place (47). Moreover, viral internalization and trafficking are known to be initiated and promoted by receptor-mediated activation of host signaling pathways, such as the PI3K/Akt signaling pathway (10, 11, 13, 15, 22, 48, 49). In the present study, we demonstrated that the PSaV Cowden strain can activate the PI3K/Akt and MEK/ERK signaling pathways at the early stage of PSaV infection. Moreover, inhibition of PSaV binding to the cell surface carbohydrate moieties or terminal sialic acids by pretreatment with NaIO_4_ or NA inhibited activation of these signaling pathways. These results suggest that binding of PSaV particles to cell surface carbohydrate receptors can activate PI3K/Akt and MEK/ERK signaling pathways to initiate and promote PSaV entry. Moreover, the infectious PSaV particles or PSaV VLPs alone, without the addition of bile acid to the medium, which is known to facilitate genome release from late endosomes (39), activated these signaling pathways at the early stage. These results further support our above hypothesis that the interaction of PSaV with the cellular carbohydrate receptors may mediate activation of these signaling pathways to initiate and promote PSaV entry. These results are similar to what has been reported for cells infected with other known viruses. For example, the interaction of envelope glycoprotein gp120 of HIV-1 with its CD4 receptor and CXCR4 coreceptor activates the PI3K/Akt and MEK/ERK signaling cascades (48, 49). In addition, activation of the PI3K/Akt signaling pathway at the early stage of HCV infection was found to be mediated through binding of the HCV E2 envelope protein with the coreceptors CD81 and claudin-1, which enhances viral entry into host cells (10). Furthermore, binding of the rotavirus outer capsid proteins VP8*, VP5*, and VP7 to their receptors or coreceptors activated both PI3K/Akt and MEK/ERK signaling pathways during the early stage of infection (22). In addition, the reduction in PSaV-induced activation of both signaling pathways by deprivation of cell membrane cholesterol was restored by the addition of soluble cholesterols, suggesting that both signaling pathways could be activated by PSaV entry through cholesterol-mediated endocytosis and vice versa, which is an ongoing area of study. To summarize, PSaV-induced early activation of PI3K/Akt and MEK/ERK signaling pathways is initiated by binding of PSaV particles to cell surface carbohydrate attachment receptors, and possibly by an entry process via cholesterol-mediated endocytosis.

A concern might still be raised about the role of PI3K/Akt and MEK/ERK activation in the PSaV entry process. Consistent with a previous report (39), we showed that PSaV particles travel from early to late endosomes during the PSaV entry process. However, inhibition of both signaling cascades by pretreatment with wortmannin and U0126, which are specific inhibitors of PI3K and MEK, respectively, sequestered PSaV particles within the early endosomes, as indicated by colocalization with the early endosome marker EEA1. This prevented PSaV trafficking to late endosomes even after 3 h postinfection, by which time PSaV particles should have been uncoated and their genomes released into the cytoplasm (33). These results suggested that PSaV-induced early activation of PI3K/Akt and MEK/ERK facilitates PSaV trafficking from early to late endosomes.

The PI3K/Akt and MEK/ERK signaling pathways are reported to participate in acidification of the late endosomes during entry by influenza A virus and the late-penetrating rotavirus strains DS-1 and NCDV (21, 22). It was shown that the participation was through direct interaction with the V-ATPase proton pump, which in turn mediates uncoating and release of the transcriptionally active form of the virus into the cytoplasm (21, 22). Since the acidification of late endosomes plays a crucial role in PSaV uncoating (45), our findings demonstrated the role of PSaV-activated pPI3K and pERK signaling molecules in the acidification of late endosomes via their interaction with the V-ATPase.

Replication of the PSaV Cowden strain in cell culture systems is extremely dependent on the presence of bile acids in the medium (31). Previous reports suggested that bile acids inhibit interferon (IFN) signaling by reducing phosphorylation of the signal transducer and activator of transcription 1 (STAT1) and/or protein kinase A (PKA) pathways, thereby facilitating PSaV replication in cells (30, 31). In contrast, our previous observation indicates that bile acids have no influence on the reduction in the STAT1-mediated signaling pathway, because PSaV replication in cell culture even in the presence of bile acids was restricted by IFN, and STAT1 and 2′,5′-oligoadenylate synthetase, which are involved in IFN-mediated signaling pathways, were activated upon PSaV infection (32). Recently, it was reported that bile acids could facilitate the escape of PSaV from late endosomes into the cytoplasm to initiate viral replication (39). In the present study, bile acid alone was also found to activate PI3K/Akt and MEK/ERK signaling pathways at the early stage in mock-infected LLC-PK cells. This suggests that in addition to facilitating PSaV escape from late endosomes (39), bile acids may also promote trafficking of PSaV particles from early to late endosomes via the activation of PI3K/Akt and MEK/ERK signaling pathways.

Currently, LLC-PK cells, derived from porcine renal proximal tubular epithelial cells, are the only permissible cells to allow the replication of PSaV strain Cowden in the presence of porcine intestinal contents or bile acids (31). These cells are similar in many respects to intestinal epithelial cells. For example, bile acids are taken up into the cells by bile acid transporters and exert various biological functions by acting on bile acid receptors (50). Similar to intestinal epithelial cells, LLC-PK cells also exhibit polarization and tight junction formation (51). Therefore, LLC-PK cells can serve as an *in vitro* model for studies of the PSaV life cycle.

In summary, we have demonstrated that PSaV-induced early activation of PI3K/Akt and MEK/ERK signaling pathways facilitates trafficking of PSaV particles from early to late endosomes. Moreover, PSaV-induced early activation of pPI3K and pERK molecules mediates the V-ATPase-dependent late endosomal acidification that is required for PSaV uncoating. The activation of these signaling pathways is triggered by the interaction of PSaV particles with cell surface attachment receptors. These results increase our comprehensive knowledge and understanding of the PSaV-host interaction during viral entry, which may provide additional information for the development of strategies for controlling or preventing PSaV infections as well as other calicivirus infections, such as human norovirus infections, which create major public health concerns.

## MATERIALS AND METHODS

### Cells and virus.

Porcine kidney epithelial cells (LLC-PK), obtained from the American Type Culture Collection (ATCC) (Manassas, VA, USA), were grown in Eagle’s minimal essential medium (EMEM) supplemented with 10% fetal bovine serum (FBS), 100 U/ml penicillin, and 100 μg/ml streptomycin. Spodoptera frugiperda ovarian cells (Sf9 cells), purchased from Gibco (Fort Worth, TX, USA), were cultured at 27°C in SF-900 II SFM medium containing 10% FBS, 100 U/ml penicillin, 100 μg/ml streptomycin, lipid medium supplementation, and 0.1% pluronic acid solution (Sigma-Aldrich, St. Louis, MO, USA).

The tissue culture-adapted PSaV Cowden strain was recovered from the full-length infectious clone pCV4A and propagated in LLC-PK cells with supplementation with 200 μM GCDCA (Sigma-Aldrich) (31). PSaV was concentrated by ultracentrifugation, and the viral titer was calculated by the method of Reed and Muench (52) and expressed as the median tissue culture infectious dose (TCID_50_) per milliliter.

### Reagents and antibodies.

Wortmannin (PI3K inhibitor) and U0126 (MEK inhibitor) were purchased from Invivogen (San Diego, CA, USA) and dissolved in dimethyl sulfoxide (DMSO). NaIO_4_, NA, methyl-beta-cyclodextrin (MβCD), soluble cholesterol, and bafilomycin A1 were obtained from Sigma-Aldrich. NaIO_4_ and NA were dissolved in phosphate-buffered saline (PBS; pH 7.2), MβCD and soluble cholesterol were dissolved in double-distilled water (DDW), and bafilomycin A1 was dissolved in DMSO. Alexa Fluor 594 (AF594) succinimidyl ester was purchased from Molecular Probes (Bedford, MA, USA) and dissolved in DMSO. GCDCA (Sigma-Aldrich) was dissolved in DDW. SlowFade Gold antifade reagent with 4′,6-diamidino-2-phenylindole (DAPI) was obtained from Molecular Probes.

Specific rabbit polyclonal antibodies against PI3K p85, pPI3K p85 (Tyr458)/p55 (Tyr199), Akt, pAkt (Ser473), p44/p42 mitogen-activated protein kinase (MAPK) (ERK1/2), and phospho-p44/p42 MAPK (pERK1/2) (Thr202/Tyr204) were purchased from Cell Signaling (Beverly, MA, USA). Rabbit anti-glyceraldehyde 3-phosphate dehydrogenase (anti-GAPDH) (FL-335) polyclonal antibody was from Santa Cruz (Dallas, TX, USA). Mouse anti-EEA1 monoclonal antibody (MAb) was obtained from BD Transduction Laboratories (Lexington, KY, USA). Mouse anti-LAMP2 and anti-V-ATPase E subunit (ATP6E) MAbs were purchased from Abcam (Cambridge, MA, USA). Hyperimmune rabbit sera raised against PSaV capsid and VPg were used in this study (43). Secondary antibodies included horseradish peroxidase (HRP)-conjugated goat anti-rabbit IgG (Cell Signaling), HRP-conjugated goat anti-mouse IgG, fluorescein isothiocyanate (FITC)-conjugated anti-mouse IgG, FITC-conjugated anti-rabbit IgG (Santa Cruz), and AF594-conjugated goat anti-mouse IgG (Life Technologies, Eugene, OR, USA).

### Recombinant VLP production.

VLPs of the PSaV Cowden strain were expressed in baculovirus-infected Sf9 cells by use of the Bac-to-Bac baculovirus expression system (Invitrogen, Waltham, MA, USA) according to the manufacturer’s instructions. Briefly, the complete sequence of PSaV Cowden strain VP1 (GenBank accession number KT922087.1) was amplified by reverse transcription-PCR (RT-PCR) with the forward primer 5ʹ-CGTGATGGAGGCGCCTGCCCCAACC-3ʹ (nucleotide positions 5136 to 5160 of the VP1 region) and the reverse primer 5ʹ-TCATCGTGAGCTGTGAATGGACCTTCC-3ʹ (nucleotide positions 6748 to 6774 of the VP1 region). Subsequently, the amplified fragment was first cloned into the pCR2.1-TOPO vector (Invitrogen) and then transformed into DH5α competent cells (Enzynomics, Daejeon, South Korea). Plasmids were purified using a GeneAll Hybrid-Q Plasmid Rapidprep kit (GeneAll, Seoul, South Korea), and the sequences were verified using an ABI system 3700 automated DNA sequencer (Applied Biosystems, Foster City, CA, USA). Using purified plasmid, the full-length cDNA copy of the capsid gene was amplified by PCR with a forward primer (5ʹ-CACAGGATCCATGGAGGCGCCTGCCCCAACC-3ʹ) containing a BamHI restriction site (underlined) and a reverse primer (5ʹ-AATCTCGAGTCATCGTGAGCTGTGAATGGACCTTCC-3ʹ) containing an XhoI restriction site (underlined). After digestion with BamHI and XhoI restriction enzymes, the amplified fragments were subcloned into the pFastBac1 baculovirus donor plasmid (Thermo Fisher Scientific, Seoul, South Korea). Recombinant baculovirus was generated by transformation of the recombinant pFastBac1 plasmid into Escherichia coli DH10Bac to produce recombinant bacmid DNA, which was then transfected into Sf9 cells by use of Cellfectin II reagent (Invitrogen). Recombinant baculovirus carrying the PSaV VP1 gene was used to infect Sf9 insect cells at an MOI of 10, and the cells were harvested at 5 to 7 days postinfection. The harvested cells were clarified by centrifugation at 3,000 × *g* for 30 min at 4°C. The VLPs were purified from cell culture supernatants by cesium chloride (CsCl) density gradient ultracentrifugation. The protein concentration of PSaV VLPs was determined by use of a bicinchoninic acid (BCA) protein assay kit (Pierce, IL, USA) according to the manufacturer’s instructions. Each purified protein was used at 10 μg/ml to test whether it could activate target signaling pathways as described below. Expression of the recombinant capsid protein was validated by electron microscopy and Western blot analysis as described below.

### TEM.

PSaV VLPs purified by CsCl density gradient ultracentrifugation were stained with 3% phosphotungstic acid (pH 7) and examined by transmission electron microscopy (TEM) (JEM-2000 FXII microscope; JEOL, USA) as described previously (36–38).

### Labeling of PSaV with AF594.

AF594 labeling of PSaV particles purified by CsCl density gradient centrifugation was performed as described elsewhere (53, 54). Briefly, purified PSaV particles (10 mg at 1 mg ml^−1^) in 0.1 M sodium bicarbonate buffer (pH 8.3) were labeled with a 1/10-fold molar concentration of AF594 succinimidyl ester (1 mg at 1 mg ml^−1^ in DMSO) according to the manufacturer’s instructions. The mixture was vortexed for 30 s and incubated for 1 h at room temperature with continuous stirring. AF594-labeled PSaV particles were repurified by CsCl density gradient centrifugation, dialyzed against virion buffer, and stored in 2-μg aliquots at –20°C. Coomassie blue staining and Western blotting of SDS-PAGE-separated AF594-labeled viral particles showed that the label was exclusively coupled to the viral protein. The infectivity of AF594-labeled PSaV in comparison to that of mock-labeled PSaV was evaluated by determining the TCID_50_ and by quantitative RT-PCR (qRT-PCR) as described below.

To calculate the number of AF594-labeled PSaV particles in each reaction mixture, the number of PSaV particles was counted as described previously (55, 56). Briefly, the above-described solution containing CsCl-purified AF594-labeled PSaV particles was mixed with an equal volume of 120-nm latex beads (Sigma-Aldrich) and then applied to the grids. The grids were stained with 3% phosphotungstic acid at pH 7 for 3 min at room temperature and observed by TEM (JEOL). The virus particles were counted along with the beads in at least 10 randomly chosen squares on the grid. The total virus count was calculated as the ratio of the virus particle number to the latex particle number and multiplied by the known latex particle concentration per milliliter.

### Chemical treatment and virus infection of LLC-PK cells.

LLC-PK cells plated on 12-well plates or 8-well chamber slides were washed twice with PBS, pH 7.4. Subsequently, the cells were mock pretreated or pretreated with a nontoxic working concentration of chemicals for 1 h at 37°C, except for NaIO_4_, which was used for 30 min at 4°C. The cytotoxic effects of the chemicals were determined using the 3-(4,5-dimethylthiazol-2-yl)-2,5-diphenyl tetrazolium bromide (MTT) assay as described elsewhere (43, 53). The cells were then infected with mock-labeled or AF594-labeled virus and used to assess PSaV-induced signaling pathways, binding and entry of PSaV by confocal microscopy, and target protein expression levels by Western blotting as described below.

### siRNA knockdown.

LLC-PK cells grown to 70% to 80% confluence were transfected with reported siRNAs against PI3K p85α or MEK (Santa Cruz) or scrambled control siRNA (Santa Cruz) (22) by use of Lipofectamine 2000 (Invitrogen) according to the manufacturer’s instructions. To optimize the knockdown efficiency, a second transfection was carried out 24 h after the first transfection, and subsequent experiments were performed 48 h later. To confirm siRNA knockdown of each target protein, LLC-PK cells treated in parallel were analyzed by Western blotting as described below.

### Virus binding assay.

The virus binding assay was performed as described elsewhere (43, 53). Briefly, mock-treated, enzyme-treated, or chemical-treated LLC-PK cells grown in 8-well chamber slides were inoculated with AF594-labeled PSaV (approximately 415 particles per cell) for 30 min at 4°C. The cells were then fixed with 4% paraformaldehyde in PBS for 15 min at room temperature and washed three times with PBS containing 0.1% newborn calf serum (PBS-NCS). The cells were mounted with SlowFade Gold antifade reagent containing 1× DAPI solution (Molecular Probes) for nuclear staining and examined using an LSM 510 confocal microscope (Carl Zeiss, Oberkochen, Germany).

### Virus internalization assay.

The virus internalization assay was performed as described elsewhere (22, 43, 53). Briefly, mock-treated, chemical-treated, or siRNA-transfected LLC-PK cells grown in 8-well chamber slides were inoculated with AF594-labeled PSaV (approximately 415 particles per cell) for 30 min at 4°C. The cells were then shifted to 37°C and incubated for the indicated times to allow viral entry to proceed. Thereafter, the cells were fixed with 4% paraformaldehyde in PBS for 15 min at room temperature, permeabilized by the addition of 0.2% Triton X-100 in PBS for 10 min at room temperature, and washed three times with PBS containing 0.1% PBS-NCS. To determine whether treatment with chemicals or transfection of siRNAs in the presence or absence of 200 μM GCDCA blocked colocalization between PSaV and endosome markers, the cells were further incubated with a MAb against EEA1 or LAMP2 (1:100 dilution) at 4°C overnight, washed twice with PBS-NCS, and then incubated with FITC-conjugated anti-mouse IgG antibody (1:100 dilution) for 1 h at room temperature. The cells were mounted for nuclear staining, and infected cells were observed with an LSM 510 confocal microscope and analyzed using LSM software. To determine the colocalization of pPI3K, pAkt, and pERK signaling molecules with V-ATPase subunit E (VATPE), LLC-PK cells were infected or not with PSaV for 30 min at 4°C. The cells were incubated for 120 min at 37°C and then fixed and permeabilized as mentioned above. The cells were further incubated with a mixture of a MAb against VATPE and rabbit polyclonal antibodies against pPI3K, pAkt, or pERK at 4°C overnight. After washing twice with PBS-NCS, the cells were incubated with AF488-conjugated donkey anti-rabbit IgG (1:100 dilution) and AF594-conjugated goat anti-mouse IgG (1:100 dilution) antibodies for 1 h at room temperature. The cells were then mounted and analyzed by confocal microscopy as mentioned above. Colocalization of the red and green signals was quantified using the ImageJ program (http://rsb.info.nih.gov/ij/) as described previously (58). Briefly, 10 images of cells treated under the conditions described above were taken by confocal microscopy and then processed, and quantification of the signals was made with a threshold of 0.03 to 1.3 µm^2^.

### Western blotting.

To determine expression levels of target proteins, Western blotting was carried out as described previously (22, 33). Briefly, mock-treated, enzyme-treated, chemical-treated, or siRNA-transfected LLC-PK cells were either mock infected or infected with PSaV (MOI of 1) in the presence or absence of 200 μM GCDCA or treated with PSaV VLPs (10 µg/ml). At the indicated time points, the cells were lysed with cell extraction buffer (Invitrogen) (10 mM Tris, pH 7.4, 100 mM NaCl, 1 mM EDTA, 1 mM EGTA, 1 mM NaF, 20 mM Na_2_P_2_O_7_, 2 mM Na_3_VO_4_, 1% Triton X-100, 10% glycerol, 0.1% SDS, 0.5% deoxycholate) supplemented with protease and phosphatase inhibitors (Roche, Basel, Switzerland) for 30 min. For detection of VLPs of the PSaV Cowden strain, recombinant baculovirus-infected Sf9 cells, their supernatant, and CsCl-purified VLPs were treated as described above. After centrifugation at 12,000 × *g* for 10 min at 4°C, the supernatants of the cell lysates were normalized for equal protein content, which was measured by use of a BCA protein assay kit (Thermo Scientific, Waltham, MA, USA). Protein samples were separated by SDS-PAGE and transferred onto nitrocellulose membranes (GE Healthcare Life Sciences, Piscataway, NJ, USA). Thereafter, the membranes were incubated for 1 h at room temperature with Tris-buffered saline containing 5% skim milk to block nonspecific reactions. The membranes were incubated overnight at 4°C with the indicated primary antibodies, followed by incubation for 1 h with HRP-labeled secondary antibody. After extensive washing, the immunoreactive bands were detected by enhanced chemiluminescence (ECL) (Dogen, Seoul, South Korea) using a Davinch-K Western imaging system (Davinch-K Co., Ltd., Seoul, South Korea).

### Immunoprecipitation assay.

Immunoprecipitation of each target protein was performed as previously described (22). Briefly, LLC-PK cells grown in 6-well plates were transfected or not with siRNAs against PI3K p85α or MEK, infected with or without the PSaV Cowden strain at an MOI of 1, and then incubated for the indicated times at 37°C. Mock-infected LLC-PK cells were incubated in parallel for the same times at 37°C. Thereafter, the cells were washed and lysed as described above. Cell lysates were precleared by incubation with protein A or G agarose beads for 30 min at 4°C. Subsequently, the precleared cell lysates were incubated with antibodies against the V-ATPase E subunit, pPI3K, pAkt, or pERK at 4°C overnight. The immune complexes were captured by incubation with protein A or G agarose beads for 1 h at 4°C, and the immunoprecipitated proteins were then evaluated by Western blotting as described above. As a control, LLC-PK cells infected with or without the PSaV Cowden strain at an MOI of 1 were lysed and prepared to check immunoprecipitation of the V-ATPase E subunit with nonphosphorylated PI3K, Akt, and ERK as described above.

### Duolink PLA.

*In situ* interactions of pPI3K, pAkt, and pERK with the V-ATPase were detected with a Duolink PLA kit (Sigma-Aldrich) as described elsewhere (22). Briefly, LLC-PK cells grown in 8-well chamber slides were infected with the PSaV Cowden strain, incubated for 60 min in the presence of 200 μM GCDCA, fixed with 4% paraformaldehyde in PBS for 15 min, and permeabilized by addition of 0.2% Triton X-100 for 10 min at room temperature. The cells were then incubated with Duolink blocking solution in a preheated humidity chamber for 30 min at 37°C, followed by incubation with a mixture of primary mouse anti-V-ATPase E subunit and rabbit anti-pPI3K, -pAkt, or -pERK antibodies overnight at 4°C. After washing twice in Duolink washing buffer A for 5 min, the cells were incubated with PLA probe anti-rabbit Minus and anti-mouse Plus secondary antibodies conjugated with oligonucleotides for 1 h in a preheated humidity chamber at 37°C. Unbound PLA probes were removed by washing twice in Duolink washing buffer A for 5 min, and then the Duolink ligation solution was applied to the slides for 30 min in a preheated humidity chamber at 37°C, followed by washing in Duolink washing buffer A twice for 2 min each time. Duolink amplification-polymerase solution was applied to the slides in a dark preheated humidity chamber for 100 min at 37°C. The slides were then washed twice in 1× Duolink washing buffer B for 10 min, followed by washing for 1 min with 0.01× Duolink washing buffer B. The cells were then mounted using Duolink *in situ* mounting medium with DAPI and observed with an LSM 510 confocal microscope (Carl Zeiss). PLA signals were recognized as red fluorescent spots.

### Virus titration by TCID_50_ assay.

Mock- or chemical-treated LLC-PK cells in 12-well plates were infected with or without PSaV at an MOI of 1 for 36 h at 37°C in the presence or absence of 200 μM GCDCA. Virus titers were determined using a TCID_50_ assay as previously described (57). Briefly, 10-fold serial dilutions of clarified supernatants obtained by three repeated freeze-thaw cycles and centrifugation from cells pretreated with or without chemicals, infected with or without PSaV, or transfected with or without siRNAs were prepared in EMEM. From these dilutions, 200 µl each was inoculated onto monolayers of LLC-PK cells grown in 96-well plates and incubated at 37°C in a 5% CO_2_ incubator. Virus titers were calculated at 6 days postinfection and expressed in TCID_50_ per milliliter by the method of Reed and Muench (52).

### Real-time RT-PCR.

To examine the infectivity of AF594-labeled PSaV or mock-labeled PSaV, real-time RT-PCR was carried out as described previously (32). Briefly, LLC-PK cells infected with AF594-labeled PSaV or mock-labeled PSaV were incubated for 36 h at 37°C in the presence of 200 μM GCDCA and harvested by freezing and thawing three times, and cell debris was collected by centrifugation at 2,469 × *g* for 10 min at 4°C. Supernatants and remaining bulk samples were collected and stored at −80°C until they were used. Total RNA was extracted using an RNeasy kit (Qiagen) according to the manufacturer’s instructions. Viral genome copy numbers were determined by one-step SYBR Green real-time RT-PCR, using a primer pair specific for the PSaV VPg gene. Each reaction mixture had a total volume of 20 μl, containing 4 μl RNA template (1 μg), 10 μl SensiFast SYBR Lo-ROX one-step mixture (Bioline, Quantace, London, United Kingdom), 0.8 μl (each) forward and reverse primers (10 pmol), 0.2 μl reverse transcriptase, 0.4 μl RiboSafe RNase inhibitor, and 3.8 μl RNase-free water. Real-time RT-PCR was performed using a Rotor-Gene real-time amplification system (Corbett Research, Mortlake, Australia) under the following conditions: reverse transcription was carried out at 50°C for 30 min, followed by activation of hot-start DNA polymerase at 95°C for 10 min and 40 cycles of 95°C for 15 s, 60°C for 30 s, and 72°C for 20 s. Quantitation of viral RNA was carried out using a standard curve derived from 10-fold serial dilutions of cRNA generated by reverse transcription of *in vitro*-transcribed control RNA (PSaV VPg gene). The threshold was automatically defined as the initial exponential phase, reflecting the highest amplification rate. A direct relationship between cycle number and the log concentration of RNA molecules initially present in the RT-qPCR reaction mixture was used to calibrate the crossing points resulting from the amplification curves for the samples.

### Statistical analysis and software.

Statistical analyses were performed on triplicate experiments by using GraphPad Prism software, version 5.03 (GraphPad Software, Inc., La Jolla, CA, USA), and one-way analysis of variance (ANOVA). *P* values of <0.05 were considered statistically significant. Figures were generated using Adobe Photoshop CS3 and Prism 6, version 6.04.
